# An mm-sized biomimetic directional microphone array for sound source localization in three dimensions

**DOI:** 10.1038/s41378-022-00389-9

**Published:** 2022-06-15

**Authors:** Ashiqur Rahaman, Byungki Kim

**Affiliations:** 1 School of Mechatronics Engineering, Korea University of Technology and Education, Cheonan, 31253 Republic of Korea; 2 Future Convergence Engineering, Korea University of Technology and Education, Cheonan, 31253 Republic of Korea

**Keywords:** Electrical and electronic engineering, Physics

## Abstract

Fly *Ormia ochracea* ears have been well-studied and mimicked to achieve subwavelength directional sensing, but their efficacy in sound source localization in three dimensions, utilizing sound from the *X*-, *Y*-, and *Z*-axes, has been less explored. This paper focuses on a mm-sized array of three *Ormia ochracea* ear-inspired piezoelectric MEMS directional microphones, where their in-plane directionality is considered a cue to demonstrate sound source localization in three dimensions. In the array, biomimetic MEMS directional microphones are positioned in a 120° angular rotation; as a result, six diaphragms out of three directional microphones keep a normal-axis relative to the sound source at six different angles in the azimuth plane starting from 0° to 360° in intervals of ±30°. In addition, the cosine-dependent horizontal component of the applied sound gives cues for *Z*-axis directional sensing. The whole array is first analytically simulated and then experimentally measured in an anechoic chamber. Both results are found to be compliant, and the angular resolution of sound source localization in three dimensions is found to be ±2° at the normal axis. The resolution at the azimuth plane is found to be ±1.28°, and the same array shows a ± 4.28° resolution when sound is varied from the elevation plane. Looking at the scope within this area combined with the presented results, this work provides a clear understanding of sound source localization in three dimensions.

## Introduction

**Fig. 1:**
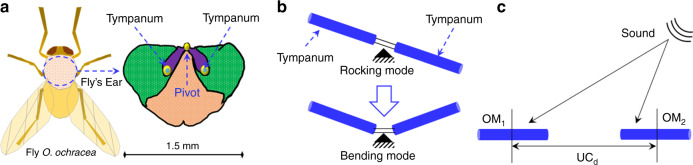
Working principle of fly-mimicking and conventional directional microphone. **a** Schematic of *O. ochracea* fly with a zoomed view of the hearing organ. **b** *O. ochracea* ear-inspired rocking mode and bending mode. **c** Conventional directional coupler using two omnidirectional microphones

Fly *Ormia ochracea*, a remarkable fly from the Tachinidae family, has an unusual hearing mechanism^1–4^. The ears, known as tympana, as shown in Fig. 1a, are spatially separated by ~450–520 µm and coupled from the middle^5–7^. This intertympanal bridge holds both tympana from each side and allows them to vibrate relative to the incoming sound^7^. The vibration of these ears generates interaural intensity difference (IID) and interaural time difference (ITD) to localize the incoming sound^5^. Since the ears are internally coupled, the vibration of the ears forms two modes: a rocking mode, where tympana show out-of-phase position, and a bending mode, utilizing the in-phase position of both ears, as shown in Fig. 1b. At these modes, IID and ITD are improved from 1 to 12 dB and 1.5 to 60 µs, respectively^8^. The improved IID and ITD allow this fly to achieve a ± 2° sound source localization (SSL) accuracy in a directional range of 30° at 5 kHz frequency^9^.

In further investigations, Miles et al. reported a spring mass damper (SMD) model of this fly where each individual ear was quantified with mass and supported by a flexible beam^5^. At critical damping, they reported that the tympanum close to the incoming sound source produces a phase difference with respect to the farthest tympanum; as a result, the acting force, the area of each tympanum multiplied by the applied sound pressure, remains the same for both tympana, but the phase difference affects both IID and ITD^5^. The applicability of their SMD model was extended by the same group, where they reported a comparative study between their SMD model and a conventional directional coupler in a stereo configuration^10^. In the stereo configuration, two omnidirectional microphones (OM1 and OM2) need to be placed in an interdistance (UC_d_) match with the applied sound wavelength, as shown in Fig. 1c. Additionally, the variation of the interdistance results in a negative impact of the directionality. For instance, if the conventional directional coupler interdistance is reduced to the ear coupling distance of the fly *O. ochracea*, the directional sensitivity can be reduced to a factor of 20log_10_(UC_d_/1.5) (see Fig. 1a for 1.5 mm)^11^. In addition to the improved directional sensitivity, this fly-mimicking directional microphone (DM) minimizes internal noise to 17.9 dBA at a reduced size^12^. These fundamental advantages of fly-mimicking directional microphones have received significant attention in realizing various acoustic applications.

The first *O. ochracea* ear-inspired MEMS directional microphone, operating in optical sensing, was reported by Gibbons et al.^13^ by utilizing the SMD model reported by Miles et al. in 1995^5^. However, controlling squeezed film damping (SFD) was the main challenge in the SMD model. Following Pandey et al.^14,15^, Ishfaque et al. reported an innovative way to control the SFD and achieve critical damping without further tuning and optimizations^16–18^. Nevertheless, the presence of a backplate was a design constraint that limited mechanical vibrations. To overcome this problem, in recent studies^19–28^, the presence of the backplate was less explored. The absence of the backplate brings two additional advantages: as almost zero SFD and cosine-dependent directionality^29,30^. The cosine-dependent directionality can be used to localize the incoming sound since it provides maximum and minimum lobes relative to the sound source position^9,19,25^. However, the directional sensing of each biomimetic DM is limited to ±90°^31^. As a result, this trend of DM provides a parabolic-shaped directional sensing rather than bidirectionality if the sound that arrives at the coupling area cannot be assumed to be zero^32^. Additionally, the sole dependency on the normal axis catalyzes noise; as a result, the SSL underlying a single *O. ochracea* fly-mimicking DM was not compliant with the fly’s accuracy, and the error rate was in the range of ±6.31°– ±7.05°, which is ~±5° lower than that of *O. ochracea*^9,25^. To overcome this problem, a well-distributed pair/array can be used to improve the capability of directional sensing as well as SSL accuracy^21^. To this end, previously reported works include a dual sensor with a 120° phase difference^19^, four orthogonally connected diaphragms^21^, three circular diaphragms spatially rotated with a 120° phase difference^33^, and a pair of circular DMs with a 90° phase difference^26,27^. However, these approaches were limited to two-dimensional (2D) SSL by focusing either on the *X*–*Y*, *X*–*Z*, or *Y*–*Z* plane separately rather than the simultaneous understanding of the *X*-, *Y*-, and *Z*-axes, as listed in Table 1. A 2D SSL leaves a limited choice in applications that suggests that the formation and demonstration of three-dimensional (3D) SSL would be an innovation within this area.

**Table 1:** Summary of previously reported SSL using fly *O. ochracea* ear-inspired MEMS directional microphone.

Refs.	Axis orientation	SSL	Microphone setup	DM dimensions (mm^2^)	Angular resolution (°)
Kuntzman et al.^9^	*X*	1D	Single	2.5 × 1.6	±6.31
Rahaman et al.^25^	X	1D	Single	1.8 × 1.1	±7.05
Wilmott et al.^19^	*X*–*Y*	2D	Pair	3.2 × 1.2	±3.4
Zhang et al.^21^	*X*–*Z*, *Y*–*Z*	2D	Pair	2.6 × 2.6	−
Rahaman et al.^26^	*X*–*Y*	2D	Pair	0.8 m (circular)	±2.92

This paper reports on 3D SSL using a mm-sized array of three identical fly *O. ochracea* ear-inspired piezoelectric MEMS DMs. Sounds from the *X*-, *Y*-, and *Z*-axes enable 3D in-plane directional sensing, which is used as a cue to perform the experimental demonstration of 3D SSL. The accuracy of 3D SSL is found to be similar to the fly at the normal-axis, which is a breakthrough within this area. We first demonstrate the aspect of modeling arrays, especially the challenges of designing a mm-sized array. We then analytically and experimentally demonstrate how an *O. ochracea* ear-inspired piezoelectric MEMS DM works and its vital acoustic characteristics, such as frequency response and directionality. Furthermore, the functionality related to 3D SSL, i.e., the directional sensing, is analytically and experimentally demonstrated by considering a sound source maintaining azimuth and elevation planes. With the understanding of a single DM, the array of three identical DMs is modeled and experimentally demonstrated, particularly by focusing on 3D directional sensing. The directional 3D sensing is used as a cue to model and demonstrate 3D SSL. Finally, we discuss the limitations and possible solutions for future research.

## Results and discussion

**Fig. 2:**
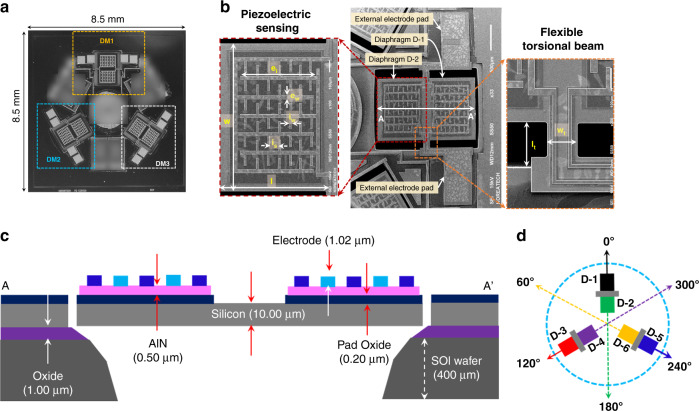
Device description. **a** Scanning electron micrograph (SEM) of the developed mm-sized array. **b** SEM of a DM showing 90° rotation from DM1 in Fig. 2a with extended view of torsional beam and piezoelectric sensing. **c** Cross-section of the fabrication, which is redrawn from the manual of PiezoMUMPs^34^. **d** Extension of the array showing the normal axis of each individual diaphragm

Figure 2a shows a scanning electron micrograph (SEM) of the developed mm-sized array that incorporates three identical *O. ochracea* ear-inspired piezoelectric MEMS DMs, denoted DM1, DM2, and DM3, fabricated on one silicon chip with dimensions of 8.5 × 8.5 mm. A zoomed view of a single DM (i.e., DM1 in 90° rotation) is shown in Fig. 2b, where two diaphragms are denoted as D-1 and D-2 and coupled from the middle, as inspired by the ear coupling mechanism of *O. ochracea* (as shown in Fig. 1a).

On top of each diaphragm, piezoelectric sensing made of aluminum nitride (AlN) and aluminum-based interdigitated electrodes (IDTs) are fabricated^34^. Upon sound application, the diaphragms start vibrating, which compresses and expands the piezoelectric sensing; as a result, an electrical signal is generated^35^. The generated electrical signals are acquired using the IDT electrodes, which are configured as *l*, *w*, *e*_l_, *e*_w_, *i*_l_, *i*_w_, and *i*_s_ are the AlN length, AlN width, main electrode length, main electrode width, IDT length, IDT width, and IDT spacing, respectively^25^. Both diaphragms (D-1 and D-2) are supported by two torsional beams, one from each side, where *l*_t_ and *w*_t_ are the length and width of torsional beam, respectively. Moreover, AA′ is the fabrication cross-section line, as shown in Fig. 2c. The fabrication is performed by using piezoelectric multiuser MEMS processes (PiezoMUMPs) following their design rules^34^, and thus the authors do not have control over the thickness of each layer, as highlighted in Fig. 2c. The value of each parameter can be found in Supplementary Table S1. Similar to DM1, diaphragms of DM2 and DM3 are denoted as D-3, D-4, D-5, and D-6, as shown in Fig. 2d. All the biomimetic DMs are positioned in a 120° angular rotation; as a result, each individual diaphragm has a normal axis at each 60° relative to the sound source in the azimuth plane, as shown in Fig. 2d. In the following sections, we discuss how the incoming sound from these six angles impacts directional sensing as well as sound source localization in three dimensions. First, we begin with a single DM to explain how it works in terms of frequency response and directionality.

### Characterization of a single biomimetic DM

#### Frequency response

**Fig. 3:**
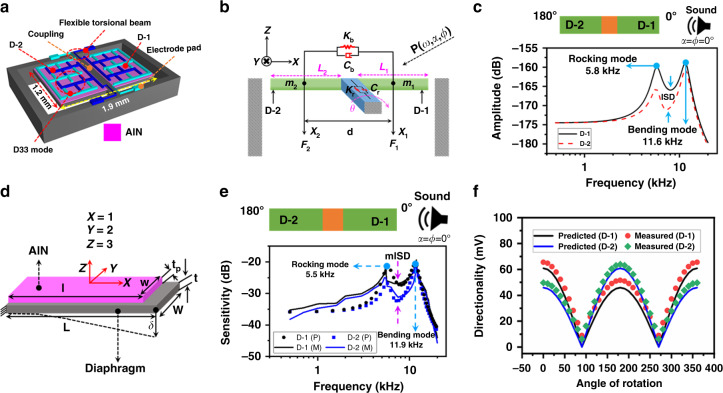
Predicted and measured acoustic sensitivity response and directionality of a single biomimetic DM. **a** Schematic of a biomimetic DM, where D-1 and D-2 are coupled from the middle by reflecting the *O. ochracea* hearing mechanism. **b** A mechanical model of Fig. 3a from the *X*–*Z* plane, where each parameter is labeled; details can be found in Supplementary Table S1. **c** Mechanical sensitivity (m/Pa) of Fig. 3b derived by Eq. 10 by considering the sound source close to diaphragm D-1 (shown at the top of Fig. 3c) at full audio frequency bands and 1 Pa sound pressure. **d** Beam deflection to analyze the electrical sensitivity (V/Pa), where *X* = 1, *Y* = 2, and *Z* = 3 show the coordinates of the piezoelectric coefficients. **e** Predicted and measured acoustic sensitivity [dB ref. 1 V at 1 Pa] considering the sound source close to diaphragm D-1 (shown at the top of Fig. 3e) with varying audio frequencies and at 1 Pa sound pressure. **f** Directionality of D-1 and D-2 in the azimuth plane, meaning that the whole biomimetic DM is rotated from 0° to 360° in the *X*–*Y* plane at 1 Pa sound pressure and 10 kHz frequency, whereas the elevation plane is fixed at 0°

Each individual DM is 1.9 × 1.2 mm (length × width) in size, and the backside is kept open, as shown in Fig. 3a. The open backside offers easier computation to model acoustic sensitivity (V/Pa). The acoustic sensitivity is a linear product of the mechanical sensitivity (m/Pa) and the electrical sensitivity (*V*/*m*). By considering the acoustic sensitivity of diaphragm D-1 as *S*_D-1_ and diaphragm D-2 as *S*_D-2_, we can define the acoustic sensitivity as

1 $$S_{{\mathrm{D}} - 1} = S_{m1} \times S_{\mathrm{e}};\,S_{{\mathrm{D}} - 2} = S_{m2} \times S_{\mathrm{e}}$$

where *S*_*m*1_ and *S*_*m*2_ are the mechanical sensitivity responses of diaphragm D-1 and diaphragm D-2, respectively. Additionally, *S*_e_ is the identical electrical sensitivity of both diaphragms.

The mechanical sensitivity (*S*_*m*1_ and *S*_*m*2_) is largely governed by the rocking and bending mode (see Fig. 1b), which depends on torsional stiffness (*K*_r_) and bending stiffness (*K*_b_), as shown in Fig. 3b, where *P* is the applied sound pressure in the *X*–*Z* direction underlying the azimuth angle (*α*) and elevation angle (*φ*). Considering the sound wavelength and device dimensions, the applied sound pressure can be expressed as^9^

2 $$p\left( {x,t} \right) = Pe\,^{j\frac{\omega }{c}x\cos \left( \alpha \right)\cos \left( \varphi \right)}e^{j\omega t}$$

where *c* is the sound velocity in air. The sound pressure distribution on the device can be expressed by the first-order Taylor series^9^. As each independent diaphragm behaves omnidirectionally in nature, the pressure component can be estimated as *P* × *e* ^*jωt*^^9^. Moreover, when the sound pressure starts interacting with the diaphragm, the diaphragm starts vibrating, which can be derived using the equation of motion considering small bending as^25^

3 $$I\ddot \theta \left( t \right) + C_r\dot \theta \left( t \right) + K_r\theta \left( t \right) = d/2 \times f_1\left( t \right) - d/2 \times f_2\left( t \right)$$

4 $$\left[ {\begin{array}{*{20}{c}} {m_1} & 0 \\ 0 & {m_2} \end{array}} \right]\left[ {\begin{array}{*{20}{c}} {\ddot x_1\left( t \right)} \\ {\ddot x_2\left( t \right)} \end{array}} \right] + \left[ {\begin{array}{*{20}{c}} {C_b} & 0 \\ 0 & {C_b} \end{array}} \right]\left[ {\begin{array}{*{20}{c}} {\dot x_1\left( t \right)} \\ {\dot x_2\left( t \right)} \end{array}} \right] + \left[ {\begin{array}{*{20}{c}} {K_b} & {K_b} \\ {K_b} & {K_b} \end{array}} \right]\left[ {\begin{array}{*{20}{c}} {x_1\left( t \right)} \\ {x_2\left( t \right)} \end{array}} \right] = \left[ {\begin{array}{*{20}{c}} {f_1\left( t \right)} \\ {f_2\left( t \right)} \end{array}} \right]$$

where *f*_1_(*t*), *f*_2_(*t*), *C*_r_, *d*, *C*_b_, and *I* are the acting force of diaphragm D-1, acting force of diaphragm D-2, damping constant at the rocking mode, interforce distance, damping constant at the bending mode, and mass moment of inertia of whole diaphragms, respectively. Equation 2 can be solved followed by the Laplace transformation as follows:^25^

5 $$\theta ( {j\omega }) = \frac{{d/2 \times \{ F_1\left( {j\omega } \right) - F_2\left( {j\omega } \right)\} }}{{I \times \left( {\omega _r^2 - \omega _{}^2 + 2\omega \omega _r^{}\zeta _r} \right)}}$$

where *θ*, *ω*, *ω*_r_, and *ξ*_r_ are the angular rotation of the diaphragm, angular frequency, rocking mode frequency, and damping ratio in rocking mode, respectively. The rocking mode frequency can be derived using torsional stiffness *K*_r_ and mass moment of inertia *I* as follows:

6 $$f_r = \frac{1}{{2\pi }} \times \sqrt {\omega _r} = \frac{1}{{2\pi }} \times \sqrt {\frac{{K_r}}{I}}$$

The formula to calculate the value of torsional stiffness and mass moment of inertia can be found in Supplementary Table S1. Similarly, Eq. 3 can be solved as follows:

7a $$\delta _1 = \frac{{F_1\left( {j\omega } \right) + \left[ {\left\{ {F_2\left( {j\omega } \right) - F_1\left( {j\omega } \right)} \right\}/\left( {\omega /\omega _b} \right)^2} \right]}}{{2m \,\times \left( {\omega _b^2 - \omega ^2 + 2j\omega \omega _b\zeta _b} \right)}}$$

7b $$\delta _2 = \frac{{F_2\left( {j\omega } \right) + \left[ {\left\{ {F_1\left( {j\omega } \right) - F_2\left( {j\omega } \right)} \right\}/\left( {\omega /\omega _b} \right)^2} \right]}}{{2m \,\times \left( {\omega _b^2 - \omega ^2 + 2j\omega \omega _b\zeta _b} \right)}}$$

where the mass (*m*), length (*L*), and acting force (*F*) caused by applied sound pressure (*P*) from azimuth (*α*) and elevation (*φ*) planes and displacement (*δ*) with subscript 1 define the parameters of diaphragm D-1, and the same parameters with subscript 2 describe diaphragm D-2. Additionally, *ω*_b_ and *ξ*_r_ are the bending mode frequency and the damping ratio at the bending mode, respectively. The bending mode frequency can be derived using torsional stiffness, *K*_b,_ and mass, m, as follows:

8 $$f_r = \frac{1}{{2\pi }} \times \sqrt {\omega _b} = \frac{1}{{2\pi }} \times \sqrt {\frac{{K_b}}{m}}$$

The formula to calculate the value of bending stiffness can be found in Supplementary Table S1. Moreover, the acting forces can be defined as the linear product of each diaphragm’s area (*A*_d_) and the sound pressure. Using Eq. 2, the force can be defined as *F*_1_ = *A*_d_ × *P* × *e*^*jωt*^ and *F*_2_ = *A*_d_ × *P* × *e*^−*jωt*^ considering the in-plane and out-of-plane vibrations of both diaphragms^9^. Eq. 5 and 7 can be utilized to derive the mechanical sensitivity of diaphragms D-1 and D-2 since Eq. 5 describes the rotational sensitivity, and Eq. 7 shows the translation sensitivity. The rotation sensitivity (*S*_θ_) can be derived as the ratio of angular rotation of the diaphragm (*θ*) and sound pressure (*P*), whereas the translation deflection (*δ*) over sound pressure is the translation sensitivity (*S*_δ_)^36^. After updating the force value, Eqs. 5 and 7 can be rewritten for the mechanical sensitivity as follows:

9a $$S_\theta = \frac{{d/2 \times A_d}}{I} \times \frac{{e^{j\omega \tau /2} - e^{ - j\omega \tau /2}}}{{\omega _r^2 - \omega ^2 + 2j\omega \omega _r\xi _r}}$$

9b $$S_{\delta 1} = \frac{{A_d}}{{2m}} \times \frac{{e^{j\omega \tau /2} + \left[ {\left\{ {e^{ - j\omega \tau /2} - e^{j\omega \tau /2}} \right\}/\left( {\omega /\omega _b} \right)^2} \right]}}{{\omega _b^2 - \omega ^2 + 2j\omega \omega _b\xi _b}}$$

9c $$S_{\delta 2} = \frac{{A_d}}{{2m}} \times \frac{{e^{ - j\omega \tau /2} + \left[ {\left\{ {e^{j\omega \tau /2} - e^{ - j\omega \tau /2}} \right\}/\left( {\omega /\omega _b} \right)^2} \right]}}{{\omega _b^2 - \omega ^2 + 2j\omega \omega _b\xi _b}}$$

Now, using Eq. 9, the mechanical sensitivity of diaphragms D-1 and D-2 can be given as

10 $$S_{m1} = S_{\delta 1} + d/2 \times S_\theta {{{\mathrm{;}}}}\,S_{m2} = S_{\delta 2} - d/2 \times S_\theta ;\,ISD = S_{m1} - S_{m2}$$

By keeping sound at *φ* = *α* = 0° (inset of Fig. 3c), the numerical analysis of mechanical sensitivity (Eq. 10) across the full audio frequencies and 1 Pa sound pressure is shown in Fig. 3c, where the peaks present resonant frequencies, such as rocking mode and bending mode, at 5.8 and 11.6 kHz, respectively.

The electrical sensitivity (*V*/*m*), on the other hand, is defined as the ratio of the generated electrical signal over the diaphragm’s displacement (*δ*) at an acting sound pressure (*P*) that can be modeled using Fig. 3d, where *t* and *t*_p_ are the thickness of the diaphragm and the AlN layer, respectively. Under short-circuit conditions, the governing equations of direct piezoelectricity are^36^

11 $$0 = C_{31}^ES_1 + C_{33}^ES_3;\,D_3 = e_{31}S_1 + e_{33}S_3$$

where *C*^E^_31_, *S*_1_, *C*^E^_33_, *S*_3_, *D*_3_, *e*_31_, and *e*_33_ are the elastic stiffness constant in the 3-1 stress–strain directions, strain component in the 1 direction, elastic stiffness constant in the 3-3 stress–strain directions, strain component in the 3 direction, electric displacement, piezoelectric constant in the 3-1 stress–strain directions, and piezoelectric constant in the 3-3 stress–strain directions, respectively. Equation 11 can be solved for *D*_3_ in the *S*_3_ beam, *D*_3_ = *e*_33f_ *S*_3_^37^. The electric displacement (*D*_3_) governs the short-circuit charge (*Q*_sc_), which can be defined as

12 $$Q_{{\mathrm{sc}}} = \frac{w}{\delta }{\int}_0^l {e_{33f}S_3dx}$$

Following the Euler–Bernoulli formula, i.e., $EI\frac{{d^4u}}{{du^4}} = f\left( x \right)$ [which can be derived as $\frac{{du\left( x \right)}}{{dx}} = \left( {\frac{{3\delta }}{{2L_s^2}}} \right)x^2 - \left( {\frac{{l\delta }}{{2L_s^3}}} \right)x^3$^37^] for the deflection in the *S*_3_ beam and charge *q* = *C*_eb_ × *V*_o_, the electrical sensitivity using the transduction ratio (*η* = *q*/*δ*) can be formulated as^35^

*η* = *q*/*δ*

13 $$\Rightarrow \frac{{3e_{33f}wL^2t}}{{4L_s^2}}\left( {a - \frac{L}{{3LL_s}}} \right) = \frac{{C_{eb}}}{\delta }; \Rightarrow S_e = \frac{{V_o}}{\delta } = \frac{{w \times t_p\left( {3l^3L - l^3} \right)}}{{4lL^3}} \times \frac{{e_{33f}}}{{C_{eb}}}\left( {V/m} \right)$$

where *C*_eb_ is the blocking capacitance which can be defined using the free capacitance generated by piezoelectric sensing^35^. In Eq. 1, the addition of mechanical sensitivity from Eq. 10 and electrical sensitivity from Eq. 13 will provide the complete formulation of the acoustic sensitivity of the device presented in Fig. 3a. The value of each individual parameter can be found in Supplementary Table S1.

After substituting Eq. 10 and Eq. 13 into Eq. 1, the acoustic sensitivity (V/Pa) of the device presented in Fig. 3a is numerically simulated, and the results are shown in Fig. 3e. Because of the close positioning of diaphragm D-1 (shown in the inset of Fig. 3e), diaphragm D-1 showed a higher response than the farthest diaphragm D-2. The validation of the numerically derived frequency response was carried out using experimental measurements, and the results are shown in Fig. 3e using a solid line. The experiment was performed in an anechoic chamber by applying 1 Pa sound pressure ~40 cm apart from the DM setup, as shown in Supplementary Fig. S1. During the whole experiment, the sound source was placed close to diaphragm D-1; a further explanation of the experimental setup can be found in the “Experimental setup” in the “Materials and Methods”. The measured resonant frequencies were found to be 5.5 and 11.9 kHz for the rocking mode and bending mode, respectively, which deviated by 5.17% and 2.52% from the analytical resonant frequencies shown in Fig. 3c. In the numerical analysis, the damping ratios were calculated using the measured frequency response. To do that, damping ratios *ξ*_r_ and *ξ*_b_ were first calculated using measured rocking and bending frequency, respectively. For the damping ratio in rocking mode, the Q-factor is derived as *Q*_r_ = *f*_rm_/Δ*f*_rm_; *f*_rm_, and Δ*f*_rm_ are the measured rocking frequency in Hz and ±3 dB bandwidth of the rocking mode, respectively. Then, the damping ratio was derived by *ξ*_r_ = *1*/(2 × *Q*_r_). Similarly, the damping ratio (*ξ*_b_) in bending mode was derived. The value of each parameter can be found in Supplementary Table S1.

#### Directionality in the azimuth plane

The sensitivity difference between diaphragms D-1 and D-2 is known as the interdiaphragm sensitivity difference (ISD) in Eq. 10 and the measured ISD (mISD) in Fig. 3e, which is an influential parameter for the directionality since this type of DM utilizes ISD to locate an incoming sound source^26^.

For better understanding, the directionality was modeled based on Supplementary Fig. S2a, where each individual diaphragm provides a higher response relative to the sound source’s position. Thus, diaphragm D-1 shows a higher response at 90° < *α* > 270°, whereas diaphragm D-2 is prominent at 90° > *α* < 270°^25^. Moreover, the directionality of this type of DM relies on the sound pressure level (SPL), frequency, and sound incidence angle, similar to an ideal acoustic-pressure gradient sensor. The formation of the directionality of each individual diaphragm can be given as^25^

14 $$\left[ \begin{array}{l}S_{{\mathrm{d1}}}\\ S_{{\mathrm{d2}}}\end{array} \right] = \left[ \begin{array}{l}S_{{\mathrm{D}} - 1}\left| {\cos \left( \alpha \right)} \right|\\ S_{{\mathrm{D}} - 2}\left| {\cos \left( \alpha \right)} \right|\end{array} \right] + \gamma \left[ \begin{array}{l}S_{{\mathrm{D}} - 2}\left| {\cos \left( \alpha \right)} \right|\\ S_{{\mathrm{D}} - 1}\left| {\cos \left( \alpha \right)} \right|\end{array} \right]{{{\mathrm{at}}}}\,\varphi = 0^ \circ$$

where *S*_d1_, *S*_d2_, *S*_D-1_, *S*_D-2_, and *γ* are the summed directionality at 90° < *α* > 270°, summed directionality at 90° > *α* < 270°, directionality of diaphragm D-1, directionality of diaphragm D-2, and delay factor, respectively. The numerical analysis of Eq. 14 is shown in Supplementary Fig. S2b–d for 3, 5, and 10 kHz frequencies of the 1 Pa sound source. Moreover, the numerical analyses were performed considering only the sound source at the azimuth angle, and the elevation angle was fixed at 0°.

**Fig. 4:**
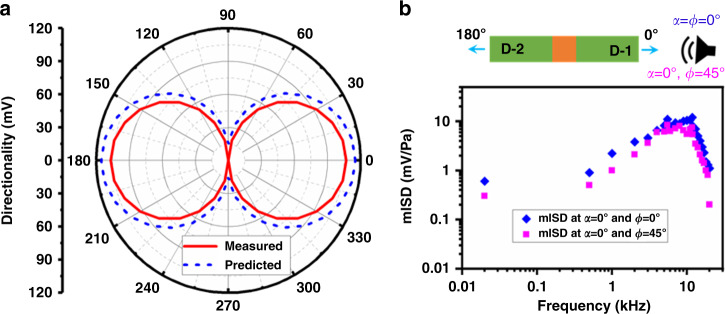
Directionality and mISD of a single biomimetic DM. **a** *Figure-8* response at 1 Pa sound pressure and 10 kHz frequency in the angular range of 0–360° in the azimuth plane. **b** The mISD at 1 Pa sound pressure at 20 Hz t0 20 kHz frequency when the sound source was positioned at (*α*, *φ*) = (0°,0°) and at (*α*, *φ*) = (0°,45°)

Figure 3f shows the predicted and measured directionality results at 10 kHz frequency and 1 Pa sound pressure. In the experimental measurement, the biomimetic DM is positioned horizontally on top of a rotation stage (shown in Supplementary Fig. S1b) and rotated about its normal axis in the azimuth plane (*α* = 0–360°) at a fixed elevation angle (*φ* = 0°)^9^. With an interval of 10°, the directionality of each individual diaphragm is measured and compared with the predicted results. Figure 3f shows that each individual diaphragm shows a higher magnitude at certain coordinates. For instance, diaphragm D-1 provides a higher magnitude at 90° < *α* > 270° compared to the other diaphragm, whereas diaphragm D-2 provides a higher response at 90° > *α* < 270°, as presented in Eq. 14. This coordinate wise response verifies the directional sensing capability of the developed DM. However, the actual bidirectionality or *figure-8* of a DM can be achieved by summing the directionality results of both diaphragms as follows:^25^

15 $$\begin{array}{l} S_{1} = (S_{\mathrm{d1}} + S_{\mathrm{d2}}) = (S_{\mathrm{D-1}} + S_{\mathrm{D-2}}) \times (1 + \gamma) \times | cos (\alpha) | \\= (S_{\mathrm{D-1}} + S_{\mathrm{D-2}}) \times \gamma^{\prime} \times | cos(\alpha) |\end{array}$$

where *S*_1_ is the summed directionality of both diaphragms at whole spans of the azimuth plane, i.e., 0–360° of a DM (say DM1 of Fig. 2d). Additionally, *γ*′ is the delay factor of both diaphragms at 0–360°. This delay was used in an ad hoc manner, meaning that this delay factor was fitted using the electrical noise of the experimental setup described by Wilmott et al.^19^. Figure 4a shows the predicted and measured *figure-8* response at 10 kHz and 1 Pa sound pressure, where it can be noted that both results are compliant with each other. In the experiment, we used a charge amplifier (SR570, Stanford Research Systems) to fit the numerical results. The charge amplifier’s sensitivity, i.e., 5 × 100 μA/V, was used as the tuning parameter, and thus we do not hold its validation beyond this sensitivity limit.

However, in Figs. 3f and 4a, the directionality was analyzed and experimentally validated by utilizing a sound source only in the azimuth plane; therefore, the impact of the sound source from the elevation plane needs to be included in realizing 3D directionality and 3D SSL. In realization, an extended experimental measurement of mISD, a form of the difference between the sensitivity responses of diaphragms D-1 and D-2, was performed, and the results are shown in Fig. 4b. Firstly, the sound source was positioned at *α* = *φ* = 0°, and the frequency response was measured. Then, the measured responses of both diaphragms were subtracted followed by Eq. 10 to achieve the mISD. In the next phase, the elevation angle was tuned to 45°; as a result, the sound source position was at (*α* = 0°, *φ* = 45°). Similarly, the frequency response of both diaphragms was measured and subtracted from each other to derive the mISD.

### Characterization of the mm-sized array

**Fig. 5:**
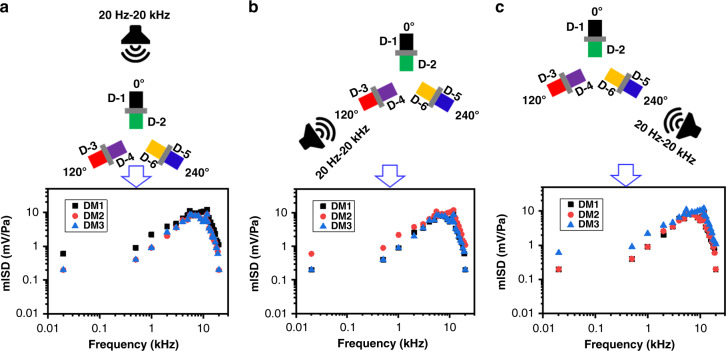
Measured ISD (mISD) response of the mm-sized array across audio frequencies by placing a 1 Pa sound source. **a** DM1 (i.e., at 0°). **b** DM2 (i.e., at 120°). **c** DM3 (i.e., at 240°)

A single DM of the developed mm-sized array (shown in Fig. 2a) is thoroughly characterized. The results are unique in terms of directionality since directionality is the cue for SSL. However, there would be room for directionality degradation due to the fabrication tolerance and interference among the DMs since all the DMs were fabricated on a single chip. Thus, prior to characterizing the directionality of the array, the mISD was measured by keeping a 1 Pa sound source at 0°, 120°, and 240°. The selection of these angles was made based on Fig. 2d, meaning that we amied to achieve the mISD of all three DMs at their normal-axis position. The results are shown in Fig. 5a–c for the sound source at 0°, 120°, and 240°, respectively. In each result, the DM close to the sound source brings higher mISD than the other two DMs.

In all cases, it can be clearly noted that the DMs show higher mISD when each of them keeps a normal-axis position relative to the sound source, which certifies their working capability as an array without being affected by each other. For instance, DM1 showed the highest mISD when the sound source was 0°, as shown in Fig. 5a, whereas DM2 and DM3 achieved the highest mISD when the sound source was positioned at 120° and 240°, respectively, as shown in Fig. 5b, c. In all cases, the formation of the mISD was followed by Eq. 10 as described for Fig. 4b.

#### Directionality in 3D

**Fig. 6:**
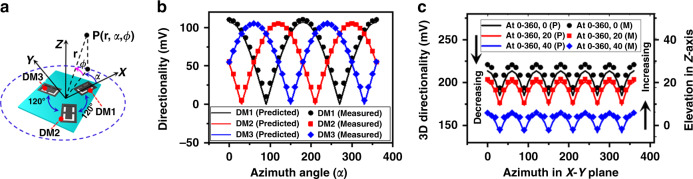
3D directionality of developed mm-sized biomimetic DM array at 1 Pa and 10 kHz frequency of sound source. **a** 3D coordinate system used in modeling of 3D directionality. **b** Directionality at azimuth plane and at a fixed elevation angle. **c** 3D directionality; where sound source was rotated simultaneously at azimuth and elevation planes, also P, and M are the predicted results and measured results, respectively

Rather than indicating only three angles, as shown in Fig. 5a–c, the formation of 3D directionality implies the simultaneous variation of sound sources from azimuth and elevation planes, as we have presented in Fig. 4b. Figure 4b shows that the improvement of the elevation angle poses a negative impact on the directionality, which is followed by the cosine dependency. This means that at higher elevation angles, the horizontal component of the applied sound decreases, which in turn minimizes phase differences and offers low mISD^38^. Thus, the elevation angle shows an inverse relationship with the mISD, which can be accounted mathematically as the cosine dependency by *cos (φ)*. Then, Eq. 15 can be rewritten using the cosine-dependent elevation angle, along with the other DMs, such as DM2 and DM3, as follows:^39^

16a $$S_1 = \left( {S_{{\mathrm{D}} - 1} + S_{{\mathrm{D}} - 2}} \right) \times \gamma {^\prime} \times \left| {\cos \left( \alpha \right)} \right| \times \left| {\cos \left( \varphi \right)} \right|$$

16b $$S_2 = \left( {S_{{\mathrm{D}} - 3} + S_{{\mathrm{D}} - 4}} \right) \times \gamma {^\prime} \times \left| {\cos \left( {\frac{{2\pi }}{3} - \alpha } \right)} \right| \times \left| {\cos \left( \varphi \right)} \right|$$

16c $$S_3 = \left( {S_{{\mathrm{D}} - 5} + S_{{\mathrm{D}} - 6}} \right) \times \gamma {^\prime} \times \left| {\cos \left( {\frac{{4\pi }}{3} - \alpha } \right)} \right| \times \left| {\cos \left( \varphi \right)} \right|$$

where *S*_1_, *S*_2_, *S*_3_ are the summed directionality of DM1 made of diaphragms D-1 and D-2, DM2 utilizing diaphragms D-3 and D-4, and DM3 for diaphragms D-5 and D-6, respectively. Moreover, in Eqs. 16b and 16c, an angular rotation between the DMs (presented in Fig. 6a) is applied. Figure 6a shows a 3D coordinate, where *P* is the applied sound pressure located at the azimuth, elevation = *α*, *φ* from distance *r*. The simplifications have been made by assuming that the whole sinusoidal acoustic pressure is converted to force^5,20,29,39^. Then, the summed directionality of the developed array underlying DM1, DM2, and DM3 can be given as^40^,

17 $$S_{{\mathrm{3}}{\mathrm{d}}} = S_1 + S_2 + S_3 = \gamma^{{\prime}{\prime}} \times \left[ \begin{array}{l}\left( {S_{{\mathrm{D}} - 1} + S_{{\mathrm{D}} - 2}} \right) \times \left| {\cos \left( \alpha \right)} \right| \times \\ \left| {\cos \left( \varphi \right)} \right| + \left( {S_{{\mathrm{D}} - 3} + S_{{\mathrm{D}} - 4}} \right) \times \left| {\cos \left( {\frac{{2\pi }}{3} - \alpha } \right)} \right| \times \\ \left| {\cos \left( \varphi \right)} \right| + \left( {S_{D - 5} + S_{D - 6}} \right) \times \left| {\cos \left( {\frac{{4\pi }}{3} - \alpha } \right)} \right| \times \left| {\cos \left( \varphi \right)} \right|\end{array} \right]$$

where *γ*″ is the delay factor of the developed array and is tuned as the charge amplifier’s sensitivity in the analytical and experimental demonstration. Additionally, *S*_3d_ of Eq. 17 is the sum of the directionality of all the directional microphones by varying the angles of incoming sound in the *X*-, *Y*-, and *Z*-axes^40^.

Supplementary Fig. S3a–c shows the simulated directionality results of the developed array at 1 Pa sound pressure and a set of frequencies, such as 3, 5, and 10 kHz, respectively. These results are derived by using Eq. 17 using parameters from Supplementary Table S1. In the analysis, the first part, second part, and third part of Eq. 17 are responsible for DM1, DM2, and DM3, respectively, as they show a 120° phase difference from one another. In all the results, the DMs show satisfactory directionality response in the sense that each diaphragm clearly shows a higher response in regard to the normal-axis position relative to the sound source. The experimental validation is performed at 1 Pa sound pressure and 10 kHz frequency.

Figure 6b shows the predicted (P) and measured (M) directionality results of the developed array by varying the sound source at the azimuth plane in a range of 0–360° and at a fixed elevation angle. In the experimental measurements, the developed array was mounted on a 1 m long rod to avoid surface reflection and rotated in the azimuth plane with 10° resolution, as shown in Supplementary Fig. S1b. The directionality of each DM was measured at each interval and compared with the predicted results. The predicted results for each DM were derived by Eq. 17. For instance, the first part of Eq. 17 was used for DM1, whereas the other two parts were used for DM2 and DM3. In all the analyses, the numerical values of each parameter were used from Supplementary Table S1. The measured results were found to be in a good match with the predicted results, where it can be noted that each diaphragm (identified D-1 to D-6) shows a maximum lobe at the normal axis, as we have expected by Fig. 2d. For instance, diaphragm D-1 showed a higher response at 0°, whereas diaphragms D-2, D-3, D-4, D-5, and D-6 showed higher responses at 180°, 120°, 300°, 240°, and 60°, respectively, as illustrated in Fig. 2d and experimentally shown in Fig. 5a–c. However, the results presented in Fig. 6b and Supplementary Fig. S3a–c are limited to the *X*–*Y* plane, meaning that the elevation plane was fixed at 0°.

Owing to this fact and utilizing the concept of Fig. 4b, Fig. 6c shows the predicted (P) and measured (M) 3D directionality at 1 Pa sound pressure and 10 kHz frequency. The predicted results (*S*_3d_) are derived by Eq. 17 by utilizing both azimuth and elevation planes. The 3D directionality was measured using the setup shown in Supplementary Fig. S1c by applying sound from both the azimuth and elevation planes. Firstly, the elevation angle in the *Z*-axis is fixed at 0°, and gradually, it was tuned to 40°. Whereas, the azimuth angles were rotated in the *X*–*Y* plane, as we did for Fig. 6b. For each rotation, the directionality of all the DMs was measured and summed together. The summed directionality was compared with the analytical result of Eq. 17. The measured directionality was found to be in good agreement with the predicted results. As previously mentioned, it should be noted that the charge amplifier’s sensitivity was used as the tuning parameter to fit the theoretical analysis, and therefore, beyond the optimized charge amplifier’s sensitivity, the results may not show this much accuracy.

Moreover, the correlation between the directionality results presented in Fig. 6b, c implies that each diaphragm showed a higher response at the normal axis, meaning that the point-to-point orientation relative to the sound source enables cosine dependency. However, the sound source with a higher elevation angle showed a negative impact that also works as a cosine dependency. In both cases, the developed array perfectly shows the directionality underlying the cosine dependency that verifies the theoretical modeling shown in Eq. 17. In the following section, our aim is to demonstrate the feasibility of the developed array for localizing a given sound source arbitrarily at different angles. The application of the discrete sound source position differs from the directionality demonstration.

#### 3D sound source localization (SSL)

By taking advantage of 3D cosine-dependent directionality, the modeling of 3D SSL can be derived using Eq. 17. At a fixed azimuth angle, the simplified form of Eq. 17 can be defined by ignoring the sine component (due to in-plane directivity) and delay factor as

18 $$S_{3{\mathrm{d}}}\left( {P,\omega ,\varphi } \right) = S_{{\mathrm{sum}}}\left( {P,\omega } \right)\cos \phi _m;\varphi _m = \cos ^{ - 1}\left( {\frac{{S_{3{\mathrm{d}}}\left( {P,\omega ,\varphi } \right)}}{{S_{{\mathrm{sum}}}\left( {P,\omega } \right)}}} \right)$$

where *S*_3d_(*P*,*ω*, *φ*) is the 3D directionality as a function of sound pressure (*P*), frequency (*ω*), and elevation angle (*φ*). Additionally, *φ*_m_ and *S*_sum_(*P*, *ω*) are the measured elevation angle in 3D space and summed response of all DMs of the developed array at a given sound, respectively. Similarly, at a fixed elevation angle, the azimuth angle can be derived as

19 $$S_{3d}\left( {P,\omega ,\varphi } \right) = S_{{\mathrm{sum}}}\left( {P,\omega } \right)\cos \alpha _m;\alpha _m = \cos ^{ - 1}\left( {\frac{{S_{3d}\left( {P,\omega ,\alpha } \right)}}{{S_{{\mathrm{sum}}}\left( {P,\omega } \right)}}} \right)$$

where *α*_m_ is the measured azimuth angle in the *X*–*Y* plane. The experimental measurement of the 3D SSL was performed based on Fig. 6a, where the applied sound source was arbitrarily varied in azimuth angles (*α*) in the *X*–*Y* plane and elevation angles (*φ*) in the *Z*-axis. At each sound source’s position, the 3D directionality (*S*_3d_(*P*,*ω*,*φ*)) as well as the simultaneous response of all three DMs (*S*_sum_(*P*, *ω*)) of the developed mm-sized array was measured. Then, Eq. 18 was used to derive the measured angle of the given sound source.

**Fig. 7:**
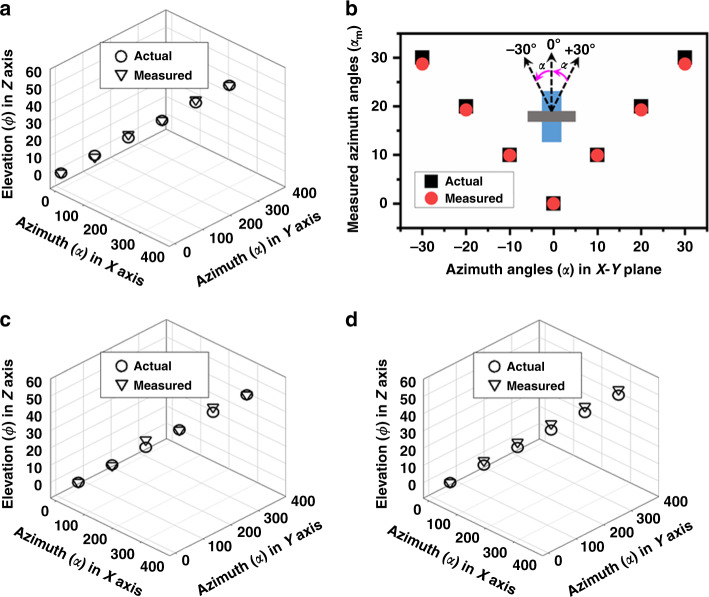
3D SSL at 10 kHz frequency and 1 Pa sound pressure. **a** Given sound source was positioned at the normal-axis relative to the developed array and varied elevation angle 0° to 50°. **b** Localization of given sound source at azimuth plane and at a fixed elevation angle. **c** 3D localization of the given sound source, where given sound source was varied in azimuth plane from 30° to 360° with an elevation from 0° to 50°. **d** 3D localization of the given sound source, where given sound source was varied in azimuth plane from 28° to 360° with an elevation from 0° to 50°

Figure 7a shows the measured angles of the given sound source comparing the actual sound source position by varying the elevation angle from 0° to 50° with an interval of 10° at 10 kHz frequency and 1 Pa sound pressure. The variation in the sound source position in the elevation plane was identically considered for each side of the diaphragm. The sound source position at the azimuth plane was tuned from 0° to 360° at an interval of 60° with primary focus to localize the given sound source using the normal axis of each diaphragm (D-1 to D-6). The measured results were compared with the actual angle of the given sound source. The maximum deviation between the measured and actual results was found to be 2° located at (*α*, *φ*) = (120°, 20°), which could be the reason for the mismatch of the sound source position and/or electrical noise of DM2 since 120° was the normal axis of diaphragm D-3^34^.

Moreover, at each localized elevation angle, the azimuth angle can be localized using the leading diaphragms, such as D-1, D-2, D-3, D-4, D-5, and D-6, at 0°/360°, 180°, 120°, 300°, 240°, and 60° in the *X*–*Y* plane (based on Fig. 2d). Figure 7b shows the localization of the given sound source at a constant elevation angle. In the measurements, at each rotation in the *X*–*Y* plane with fixed elevation, the 3D directionality (*S*_3d_(*P*, *ω* ,*α*)) of the subjected diaphragm is measured and used in Eq. 19. As shown in Fig. 7b, the maximum deviation was found to be 1.28° at ±30°. The fundamental reason for the higher deviation is the influence of other diaphragms at ±30°. Furthermore, how this deviation at ±30° biases the 3D localization of the elevation angle was measured in a combination of (*α*, *φ*) = (30°, 0°), (90°, 10°), (150°, 20°), (210°, 30°), (270°, 40°), and (330°, 50°) for six iterations. The measured and compared results are presented in Fig. 7c, where the maximum deviation was 4.28° at (*α*, *φ*) = (150°, 20°), which is still an influence of DM2. Thus, the authors believe that this could be the fabrication toleration of DM2.

Furthermore, for a better understanding of interdiaphragm interference at ±30°, the 3D SSL was measured starting with 28° with the combination of (*α*, *φ*) = (28°, 0°), (88°, 10°), (148°, 20°), (208°, 30°), (268°, 40°), and (328°, 50°) for six iterations to demonstrate how well the array can work relative to the intersection between diaphragms. The results are shown in Fig. 7d; as the localization angle is minimized by more than 1° in Fig. 7c, the accuracy was improved from 4.28° to 3.69°. In addition to the influence of the intersection between the diaphragms and fabrication tolerance, the deviations could be the reason for the tolerance of the rotational stage in the *X*–*Y* plane and manual adjustment in the *Z*-axis^26,27^.

## Conclusion

The primary intent of this work was to demonstrate 3D SSL using an mm-sized array of three identical fly *O. ochracea* ear-inspired piezoelectric MEMS directional microphones. The feasibility study was performed starting with a single biomimetic DM down to the frequency response and directionality. The results were compared with a complete analytical model of the developed DM. The results were found to be in compliance with the analytical results by using a tuning parameter, i.e., the sensitivity of the charge amplifier. Then, the directionality of the developed mm-sized array was measured and validated with theoretical analysis. Our aim for 3D directionality was to show how the developed array works relative to a sound source in the whole azimuth plane and a fixed elevation plane. The directionality showed cosine dependency and allowed us to model 3D SSL. In the 3D SSL measurement, we used the discrete position of the given sound source to understand the developed array’s sound localization capability. The developed array showed SSL accuracy similar to that of *O. ochracea* on the normal axis; however, the accuracy degradation began when we placed the sound source slightly far from the normal axis. This work uncovers novel aspects, such as the first representation of 3D SSL using an *O. ochracea* ear-inspired piezoelectric MEMS directional microphone, a complete physical model of this trend of directional microphone underlying the piezoelectric sensing, and the highest SSL accuracy thus far. Moreover, it should be noted that the validation of this work was performed at a 10 kHz frequency since analytical and measured modal frequencies showed a deviation. Thus, the selection of 10 kHz should not be considered only the operating frequency of the developed array, as Supplementary Fig. S3 shows its working capability with other frequency bands.

In addition to the unique advancements presented in this work, several limitations were found. Firstly, the tuning parameter is based on the external electrical circuit. This means that the results presented in this work are limited to a specific sensitivity of the charge amplifier, which may impose a challenge in the practical realization of the developed array. Secondly, 3D SSL was measured using only the angular position of the given sound source, and the distance was not measured, which is beyond the scope of this work. Thirdly, this DM trend utilizes ISD for directionality, which is challenging if the given sound source frequency is changed^32^. This is the greatest drawback of this DM trend. Solutions to these limitations will be the focus of future work.

## Materials and methods

### Experimental setup

The acoustic characterizations presented in this paper were carried out using two different experimental setups. Moreover, in both setups, the developed array shown in Fig. S1a was used identically. For instance, the frequency response and directionality in the *X*–*Y* plane of a single DM are shown in Fig. S1b. The same setup was extended to the measurements of the developed array in the *X*–*Y* plane. The experimental measurements in 3D using simultaneous variation of the given sound source from azimuth and elevation planes were performed by the experimental setup shown in Fig. S1c.

The installation of the experimental setup in an anechoic chamber began with the connection of the developed array. At first, the developed array was placed onto a custom-made printed circuit board (PCB). Then, the external electrode pads of each directional microphone and the PCB electrode pad were connected using a microwire bonding machine (K&C 4522, Kulicke & Soffa), as shown in Fig. S1a. Then, the PCB along with the developed array was mounted on a rotation stage (PRM1Z8, Thorlabs), which was controlled using a DC motor (KDC101, Thorlabs). The rotational stage was incorporated to accurately measure the directionality, as shown in the inset of Fig. S1b. Then, the rotational stage along with the developed array was mounted on a 1 m long beam to avoid surface reflection by the applied sound pressure, as shown in the inset of Fig. S1a, b^25,35,37^. Finally, electromagnetic shielding was used to cover the developed array to avoid interference from acoustic signals^37^.

Once the microphone setup was completed, a charge amplifier (SR 570, Stanford Research Systems) was connected just after the developed array. The device sensitivity of the charge amplifier was used as the tuning parameter to fit the theoretical results. The tuning device was 5 × 100 µA/V, and the experimental results were validated only for this sensitivity parameter. Then, the response of the charge amplifier was processed and recorded using a lock-in amplifier (SR830, Stanford Research Systems), as shown in Fig. S1b.

Furthermore, the sound was generated using a function generator (DS345, Stanford Research Systems), and the generated sound was applied by a speaker (BOS-5000 series). Same function generator was used to sync the lock-in amplifier’s frequency. To calibrate the applied sound pressure, a reference microphone (pressure-field microphone, Digital sound level meter, DL1351) was placed vertically near the developed array, as shown in Fig. S1b. The SPL of the applied sound was measured using a reference microphone and verified using a 1/8” pressure-field microphone (B&K 4138) placed vertically near the developed array^22^. The positioning of the sound source in Fig. S1b was limited to the *X*–*Y* plane that can cover the azimuth plane. Thus, an extension of Fig. S1b is accounted for, as shown in Fig. S1c, where a new sound source is added to the elevation plane. Using the experimental setup shown in Fig. S1c, the 3D measurements, such as mISD (Fig. 4a), directionality (Fig. 6c) and SSL (Fig. 7a, c, d), were performed.

## Supplementary information

Supplementary information

## Data Availability

The supporting information has been submitted, and extended data can be made available upon reasonable request from the corresponding author (Dr. Byungki Kim, email: byungki.kim@koreatech.ac.kr).
