# Supplementary material for: An mm-sized biomimetic directional microphone array for sound source localization in three dimensions
Source: Microsyst Nanoeng. 2022 Jun 15;8:66. doi: 10.1038/s41378-022-00389-9 (PMC9200786; doi:10.1038/s41378-022-00389-9)
Supplement: Supplementary file 1 — Supplementary information [file 41378_2022_389_MOESM1_ESM.docx]

**Materials and methods**

**Supplementary Table S1**

**Table S1.** Designed and simulated parameters of this work.

| **Symbol** | **Description** | **Value** | **Unit** |
| --- | --- | --- | --- |
| P | Applied acoustic pressure | 1 | Pa |
| w_t_ | Torsional beam width | 130 | μm |
| l_t_ | Torsional beam length | 150 | μm |
| t | Thickness | 10.7 | μm |
| J=βw_t_t^3^ | Torsional constant **^22^** | 43290 | μm^4^ |
| υ | Poisson’s ratio | 0.28 | N/A |
| E | Young’s modulus | 160 | GPa |
| $G=\frac{E}{2(1+\nu)}$ | Shear modulus **^22^** | 6.25×10^3^ | kg/μms^2^ |
| $K_{r}=\frac{J\times G}{l_{t}}$ | Torsional stiffness **^22^** | 6644723 | kg. μm^2^/s^2^ |
| m | Mass | 5.26×10^-8^ | kg |
| L=L_1_=L_2_ | Length of each diaphragm | 880 | μm |
| I | Mass moment of inertia | 1.35×10^-2^ | kg. μm^2^ |
| $f_{r}=\frac{1}{2\pi}\times\sqrt{\frac{K_{r}}{I}}$ | Rocking frequency in Hz | 5773 | Hz |
| W | Width of each diaphragm | 1200 | μm |
| $K_{b}=\frac{EWt^{3}}{L^{3}}$ | Bending stiffness **^22^** | 281.74 | kg. m^2^/s^2^ |
| $f_{b}=\frac{1}{2\pi}\times\sqrt{\frac{K_{b}}{m}}$ | Bending frequency in Hz | 11648 | Hz |
| d | Interdiaphragm distance | 1010 | μm |
| A_d_ | Area of each diaphragm | 1.056×10^-6^ | m^2^ |
| f_rm_ | Measured rocking mode frequency | 5490 | Hz |
| Δf_rm_ | ±3 dB bandwidth at rocking mode | 2053 | Hz |
| Q_r_=f_rm_/Δf_rm_ | Q-factor at rocking mode | 2.67 | N/A |
| ξ_r_ = 1/(2Q_r_) | Damping ratio at rocking mode **^25^** | 0.1872 | N/A |
| f_bm_ | Measured bending mode frequency | 11870 | Hz |
| Δf_bm_ | ±3 dB bandwidth at bending mode | 2461 | Hz |
| Q_r_=f_rm_/Δf_rm_ | Q-factor at bending mode | 4.82 | N/A |
| ξ_b_= 1/(2Q_b_) | Damping ratio at bending mode **^25^** | 0.1036 | N/A |
| C_eb_ | Blocking capacitance | 65.6 | fF |
| t_p_ | AlN thickness | 0.5 | μm |
| e_l_ | Length of main electrode | 650 | μm |
| e_w_ | Width of main electrode | 30 | μm |
| i_l_ | IDT length | 90 | μm |
| i_w_ | IDT width | 20 | μm |
| i_s_ | IDT spacing | 70 | μm |
| l | Length of AlN layer | 750 | μm |
| w | Width of AlN layer | 1010 | μm |

**Experimental setup**

The acoustic characterizations presented in this paper were carried out using two different experimental setups. Moreover, in both setups, the developed array shown in **Figure S1a** was used identically. For instance, the frequency response and directionality in the X-Y plane of a single DM are shown in **Figure S1b**. The same setup was extended to the measurements of the developed array in the X-Y plane. The experimental measurements in 3D using simultaneous variation of the given sound source from azimuth and elevation planes were performed by the experimental setup shown in **Figure S1c**.


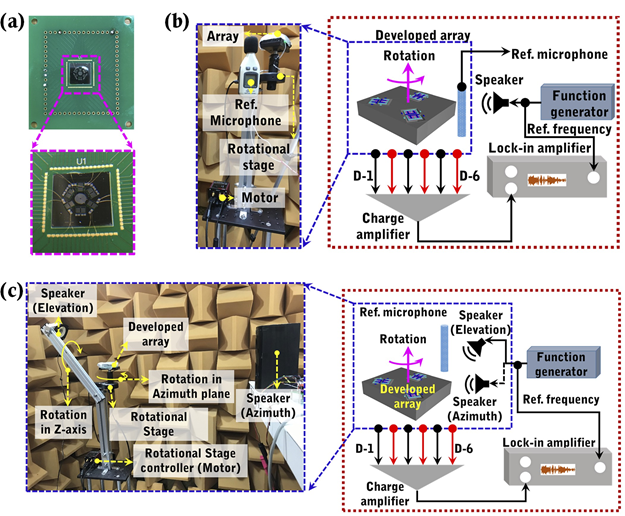


**Figure S1.** Experimental setup in an anechoic chamber. **a.** Custom PCB along with the developed array. **b.** Experimental setup to perform the measurements by varying sound source at the azimuth plane. **c.** Experimental setup to measure 3D directionality and 3D SSL by varying sound source simultaneously at azimuth and elevation planes.

The installation of the experimental setup in an anechoic chamber began with the connection of the developed array. At first, the developed array was placed onto a custom-made printed circuit board (PCB). Then, the external electrode pads of each directional microphone and the PCB electrode pad were connected using a microwire bonding machine (K&C 4522, Kulicke & Soffa), as shown in **Figure S1a**. Then, the PCB along with the developed array was mounted on a rotation stage (PRM1Z8, Thorlabs), which was controlled using a DC motor (KDC101, Thorlabs). The rotational stage was incorporated to accurately measure the directionality, as shown in the inset of **Figure S1b**. Then, the rotational stage along with the developed array was mounted on a 1 m long beam to avoid surface reflection by the applied sound pressure, as shown in the inset of **Figure S1a-b ^25, 35, 37^**. Finally, electromagnetic shielding was used to cover the developed array to avoid interference from acoustic signals **^37^**.

Once the microphone setup was completed, a charge amplifier (SR 570, Stanford Research Systems) was connected just after the developed array. The device sensitivity of the charge amplifier was used as the tuning parameter to fit the theoretical results. The tuning device was 5×100 µA/V, and the experimental results were validated only for this sensitivity parameter. Then, the response of the charge amplifier was processed and recorded using a lock-in amplifier (SR830, Stanford Research Systems), as shown in **Figure S1b**.

Furthermore, the sound was generated using a function generator (DS345, Stanford Research Systems), and the generated sound was applied by a speaker (BOS-5000 series). Same function generator was used to sync the lock-in amplifier’s frequency. To calibrate the applied sound pressure, a digital reference microphone (pressure-field microphone, Digital sound level meter, DL1351) was placed vertically near the developed array, as shown in **Figure S1b**. The SPL of the applied sound was measured using a digital reference microphone and verified using a 1/8'' pressure field microphone (B&K 4138) placed vertically near the developed array **^22^**. The positioning of the sound source in **Figure S1b** was limited to the X–Y plane that can cover the azimuth plane. Thus, an extension of **Figure S1b** is accounted for, as shown in **Figure S1c**, where a new sound source is added to the elevation plane. Using the experimental setup shown in **Figure S1c**, the 3D measurements, such as mISD (**Figure 4a**), directionality (**Figure 6c**) and SSL (**Figure 7a, c, d**), were performed.

**Supplementary Figure S2**

**
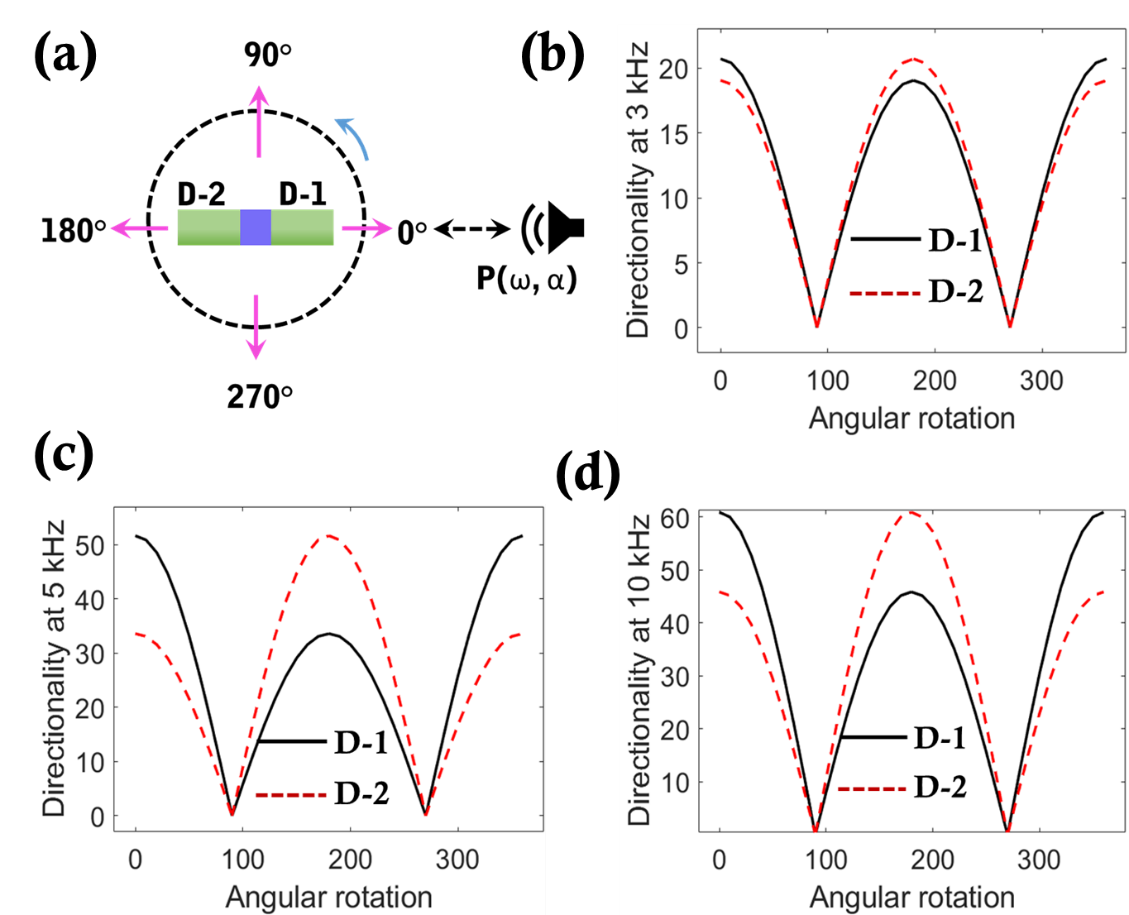
**

**Figure S2.** Model and simulated directionality result of a single biomimetic DM at the azimuth plane in a range of 0°–360° derived by **Equation 14**. **a.** Schematic. **b.** Directionality at 1 Pa and 3 kHz frequency sound source. **c.** Directionality at 1 Pa and 5 kHz frequency sound source. **d.** Directionality at 1 Pa and 10 kHz frequency sound source.

**Supplementary Figure S2**


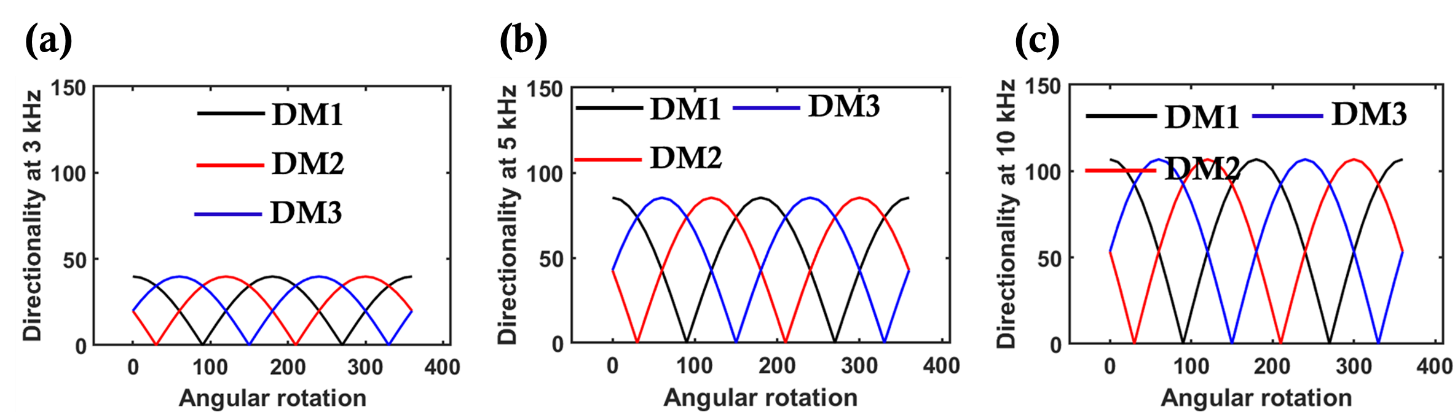


**Figure S3.** Analytically simulated directionality results of the developed mm-sized array derived by **Equation 17** by varying sound source simultaneously at azimuth (0°–360°) and elevation (φ =0°) planes at 1 Pa sound pressure and: **a.** at 3 kHz. **b.** at 5 kHz. **c.** at 10 kHz.

**Data availability statements**

The supporting information has been submitted, and extended data can be made available upon reasonable request from the corresponding author (Dr. Byungki Kim, email: byungki.kim@koreatech.ac.kr).

**Acknowledgments**

This work was supported by grants NRF–2018R1A6A1A03025526 under the Priority Research Program through the National Research Foundation of Korea (NRF) under the Ministry of Education, and in part by NRF– 2021R1A2C1004540 under the Ministry of Science and ICT, and in part by the BK-21 Four program through National Research Foundation of Korea (NRF) under Ministry of Education.

**Conflicts of interest**

The author(s) declare no competing interests.

**Contributions**

A.R. carried out all modeling, data acquisition, and wrote the original draft. B.K. supervised all studies. Both authors discussed the results and reviewed the manuscript.

**References**

1. Cade, W. Acoustically orienting parasitoids: fly phonotaxis to cricket song. ***Sci.*** 190, 1312–1313 (1975).
2. Narins, P. M. In a fly’s ear. ***Nature*** 410, 644–645 (2001).
3. Mason, A. C., Oshinsky, M. L. & Hoy, R. R. Hyperacute directional hearing in a microscale auditory system. ***Nature*** 410, 686–690 (2001).
4. Robson, D. Mic based on fly ear pinpoints sound exactly. ***New Sci.*** 197, 26 (2008).
5. Miles, R., Robert, D. & Hoy, R. Mechanically coupled ears for directional hearing in the parasitoid fly *Ormia ochracea*. ***The J. Acoust. Soc. Am.*** 98, 3059–3070 (1995).
6. Robert, D., Amoroso, J. & Hoy, R. R. The evolutionary convergence of hearing in a parasitoid fly and its cricket host. ***Sci***. 258, 1135–1137 (1992).
7. Robert, D., Miles, R. & Hoy, R. Directional hearing by mechanical coupling in the parasitoid fly *Ormia ochracea*. ***J. Comp. Physiol. A*** 179, 29–44 (1996).
8. Liu, H., Currano, L., Gee, D., Helms, T. & Yu, M. Understanding and mimicking the dual optimality of the fly ear. ***Sci. reports*** 3, 1–6 (2013).
9. Kuntzman, M. L. & Hall, N. A. Sound source localization inspired by the ears of the *Ormia ochracea*. ***Appl. Phys. Lett.*** 105, 033701 (2014).
10. Miles, R. Comparisons of the performance of Knowles hearing aid microphones to that of the Binghamton *Ormia*-inspired gradient microphone. ***State Univ. New York at Binghamton, Binghamton***, NY (2015).
11. Miles, R. & Hoy, R. The development of a biologically-inspired directional microphone for hearing aids. ***Audiol. Neurotol.*** 11, 86–94 (2006).
12. Miles, R. et al. A low-noise differential microphone inspired by the ears of the parasitoid fly *Ormia ochracea*. ***The J. Acoust. Soc. Am.*** 125, 2013–2026 (2009).
13. Gibbons, C. & Miles, R. Design of a biomimetic directional microphone diaphragm. ***Proc. IMECE, Int. Mech. Eng. Congr. Expo***. (2000).
14. Pandey, A. K., Pratap, R. & Chau, F. S. Analytical solution of the modified Reynolds equation for squeeze film damping in perforated mems structures. ***Sensors Actuators A: Phys.*** 135, 839–848 (2007).
15. Pandey, A. K. & Pratap, R. A comparative study of analytical squeeze film damping models in rigid rectangular perforated mems structures with experimental results. ***Microfluid. Nanofluidics*** 4, 205–218 (2008).
16. Ishfaque, A. & Kim, B. Analytical modeling of squeeze air film damping of biomimetic MEMS directional microphone. ***J. Sound Vib.*** 375, 422–435 (2016).
17. Ishfaque, A. & Kim, B. Squeeze film damping analysis of biomimetic micromachined microphone for sound source localization. ***Sensors Actuators A: Phys.*** 250, 60–70 (2016).
18. Ishfaque, A. & Kim, B. Analytical solution for squeeze film damping of MEMS perforated circular plates using Green’s function. ***Nonlinear Dyn.*** 87, 1603–1616 (2017).
19. Wilmott, D., Alves, F. & Karunasiri, G. Bio-inspired miniature direction finding acoustic sensor. ***Sci. reports*** 6, 29957 (2016).
20. Zhang, Y. *et al.* A low-frequency dual-band operational microphone mimicking the hearing property of *Ormia ochracea*. ***J. Microelectromechanical Syst.*** 27, 667–676 (2018).
21. Zhang, Y. *et al.* A MEMS microphone inspired by *Ormia* for spatial sound detection. ***In 2018 IEEE Micro Electro Mechanical Systems (MEMS)***, 253–256 (IEEE, 2018).
22. Rahaman, A., Ishfaque, A., Jung, H. & Kim, B. Bio-inspired rectangular shaped piezoelectric MEMS directional microphone. ***IEEE Sensors J.*** 19, 88–96 (2018).
23. Ishfaque, A., Rahaman, A. & Kim, B. Bioinspired low noise circular-shaped MEMS directional microphone. ***J. Micro/Nanolithography, MEMS, MOEMS*** 18, 010501 (2019).
24. Rahaman, A., Ishfaque, A. & Kim, B. Effect of torsional beam length on acoustic functionalities of bio-inspired piezoelectric MEMS directional microphone. ***IEEE Sensors J.*** 19, 6046–6055 (2019).
25. Rahaman, A. & Kim, B. Sound source localization by *Ormia ochracea* inspired low–noise piezoelectric MEMS directional microphone. ***Sci. Reports*** 10, 1–10 (2020).
26. Rahaman, A. & Kim, B. Sound source localization in 2D using a pair of bio–inspired MEMS directional microphones. ***IEEE Sensors J.*** 21, 1369–1377 (2021).
27. Rahaman, A. & Kim, B. Fly-inspired mems directional acoustic sensor for sound source direction. ***In 2019 20th International Conference on Solid-State Sensors, Actuators and Microsystems & Eurosensors XXXIII (TRANSDUCERS & EUROSENSORS XXXIII)***, 905–908 (IEEE, 2019).
28. Rahaman, A., Jung, H. & Kim, B. Coupled d33 mode-based high performing bio-inspired piezoelectric MEMS directional microphone. ***Appl. Sci.*** 11, 1305 (2021).
29. Touse, M. *et al.* Fabrication of a microelectromechanical directional sound sensor with electronic readout using comb fingers. ***Appl. Phys. Lett.*** 96, 173701 (2010).
30. Touse, M., Sinibaldi, J. & Karunasiri, G. MEMS directional sound sensor with simultaneous detection of two frequency bands. ***In SENSORS, 2010 IEEE***, 2422–2425 (IEEE, 2010).
31. Lee, T., Nomura, T., Su, X. & Iizuka, H. Fano-Like Acoustic Resonance for Subwavelength Directional Sensing: 0–360 Degree Measurement. ***Adv. Sci.*** 7, 1903101 (2020).
32. Zhang, Y., Reid, A. & Windmill, J. F. C. Insect-inspired acoustic micro-sensors. ***Curr. opinion insect science*** 30, 33–38 (2018).
33. Lisiewski, A., Liu, H., Yu, M., Currano, L. & Gee, D. Fly-ear inspired micro-sensor for sound source localization in two dimensions. ***The J. Acoust. Soc. Am.*** 129, EL166–EL171 (2011).
34. Cowen, A., Hames, G., Glukh, K. & Hardy, B. PiezoMUMPs design handbook. MEMSCAP Inc 1, DOI: http://www.memscapinc.com (2014).
35. Rahaman, A., Park, C. H. & Kim, B. Design and characterization of a MEMS piezoelectric acoustic sensor with the enhanced signal–to–noise ratio. ***Sensors Actuators A: Phys***. 112087 (2020).
36. Kuntzman, M. L., Gloria Lee, J., Hewa-Kasakarage, N. N., Kim, D. & Hall, N. A. Micromachined piezoelectric microphones with in-plane directivity. ***Appl. physics letters*** 102, 054109 (2013).
37. Kuntzman, M. L. Micromachined in-plane acoustic pressure gradient sensors. ***Ph.D. thesis***, The University of Texas at Austin, Texas (2014).
38. Lisiewski, A. P. Fly-Ear Inspired Miniature Sensor System for Two-Dimensional Sound Source Localization. ***Ph.D. thesis***, University of Maryland, College Park (2011).
39. Liu, H., Currano, L., Gee, D., Yang, B. & Yu, M. Fly-ear inspired acoustic sensors for gunshot localization. ***In Bio-Inspired/Biomimetic Sensor Technologies and Applications***, vol. 7321, 73210A (International Society for Optics and Photonics, 2009).

Merimaa, J. Applications of a 3-D microphone array. ***In Audio Engineering Society Convention*** 112 (Au
